# Genetic Architecture and Candidate Genes for Pubescence Length and Density and Its Relationship With Resistance to Common Cutworm in Soybean

**DOI:** 10.3389/fpls.2021.771850

**Published:** 2022-01-07

**Authors:** Yawei Li, Li Chu, Xiaofeng Liu, Nannan Zhang, Yufei Xu, Benjamin Karikari, Yu Wang, Fangguo Chang, Zexinan Liu, Lianmei Tan, Han Yue, Guangnan Xing, Tuanjie Zhao

**Affiliations:** ^1^Soybean Research Institute/MARA National Center for Soybean Improvement/MARA Key Laboratory of Biology and Genetic Improvement of Soybean/National Key Laboratory for Crop Genetics and Germplasm Enhancement/Jiangsu Collaborative Innovation Center for Modern Crop Production, Nanjing Agricultural University, Nanjing, China; ^2^Department of Crop Science, Faculty of Agriculture, Food and Consumer Sciences, University for Development Studies, Tamale, Ghana

**Keywords:** soybean, QTL mapping, pubescence length and density, candidate gene, resistance to common cutworm

## Abstract

Soybean pubescence plays an important role in insect resistance, drought tolerance, and other stresses. Hence, a deep understanding of the molecular mechanism underlying pubescence is a prerequisite to a deeper understanding of insect resistance and drought tolerance. In the present study, quantitative trait loci (QTL) mapping of pubescence traits was performed using a high-density inter-specific linkage map of one recombinant inbred line (RIL) population, designated NJRINP. It was observed that pubescence length (PL) was negatively correlated with pubescence density (PD). A total of 10 and 9 QTLs distributed on six and five chromosomes were identified with phenotypic variance (PV) of 3.0–9.9% and 0.8–15.8% for PL and PD, respectively, out of which, eight and five were novel. Most decreased PL (8 of 10) and increased PD (8 of 9) alleles were from the wild soybean *PI 342618B*. Based on gene annotation, Protein ANalysis THrough Evolutionary Relationships and literature search, 21 and 12 candidate genes were identified related to PL and PD, respectively. In addition, *Glyma.12G187200* from major QTLs *qPL-12-1* and *qPD-12-2*, was identified as *Ps* (sparse pubescence) before, having an expression level of fivefold greater in *NN 86-4* than in *PI 342618B*, hence it might be the candidate gene that is conferring both PL and PD. Based on gene expression and cluster analysis, three and four genes were considered as the important candidate genes of PL and PD, respectively. Besides, leaves with short and dense (SD) pubescence, which are similar to the wild soybean pubescence morphology, had the highest resistance to common cutworm (CCW) in soybean. In conclusion, the findings in the present study provide a better understanding of genetic basis and candidate genes information of PL and PD and the relationship with resistance to CCW in soybean.

## Introduction

Plants are sessile in nature, therefore, they are exposed to various abiotic and biotic stresses, such as drought, chilling injury, insects, and diseases attack (Zhu, 2016). Pubescence in plants offers the opportunity for them to withstand a number of stresses. Trichomes, the epidermal outgrowths with single-cell or multicellular structures covering most aerial plant tissues, are present in the enormous number of plant species (Huchelmann et al., 2017), playing extremely important roles in plant growth and development, such as protecting plants from herbivore attacks and pathogens (Hanley et al., 2007; Bickford, 2016), protecting against damaging ultraviolet (UV) radiation, avoiding excessive transpiration (Manetas, 2003; Pattanaik et al., 2014; Matias-Hernandez et al., 2016), and so on. In addition, the pubescence of single cell could be used as a model to research cell differentiation and fate (Hulskamp, 2004; Serna, 2004; Yang and Ye, 2013). Hence, it is of great significance to have a deeper understanding of the genetic basis and regulatory network of pubescence development.

In soybean, pubescence is single stalked and covers almost all aerial organs except cotyledons and hypocotyls (Liu et al., 2020). Previous research proved that pubescence density (PD) of soybean is related to some agronomic traits, such as yield, insect resistance, plant height, and evapotranspiration rates (Ghorashy et al., 1971; Singh et al., 1971; Specht et al., 1985; Clawson et al., 1986; Lam and Pedigo, 2001). Insect damage is one of the most serious stresses in crops, such as soybean, in view of this, many farmers use biotechnology-engineered crops, insecticidal seed treatments, soil-applied insecticides, and foliar sprays to manage insects (Chang and Hartman, 2017; Hurley and Mitchell, 2020). Pubescence on the surface of soybean plays an indispensable role in insect resistance (Oki et al., 2012). In the past, many researchers have studied the relationship between different pubescence morphology and insect resistance. For instance, dense pubescence and extra-dense pubescence can provide resistance to soybean mosaic virus by limiting the spread of aphid vectors (Gunasinghe et al., 1988). It has also been found that regardless of PD, soybean with long and erect pubescence has higher resistance to potato leafhopper (PLH) than their counterparts with short and close pubescence (Turnipseed, 1977). In addition, soybean variety “Camp” with low trichome density on the abaxial surface of soybean trifoliate was less attractive to *Megacopta cribraria*, and further research revealed that “Camp” also exhibited antibiosis by suppressing nymph development (Lahiri et al., 2020a,b). To date, a number of quantitative trait loci (QTL) for insect resistance have been reported in soybean and some colocalized with pubescence-related traits, for example, the PLH locus is close but distinct to a PD QTL on the chromosome (Chr) 12 (Chang and Hartman, 2017). The pubescence QTLs on Chr07 and Chr12 were located near the antixenosis resistance QTLs: *qRslx1* and *qRslx2*, respectively (Oki et al., 2012).

Many statistical methods have been developed for QTL detection with composite interval mapping (CIM) as one of the most widely used methods. CIM was proposed to combine interval mapping and multiple regression analysis (Zeng, 1993). Molecular markers were used to limit genetic background effects, thereby, reducing false positives and improving mapping accuracy. However, there are some limitations, among them including its inability to analyze epistatic QTLs, additive by additive, and additive by environment interactions. To resolve the above shortcomings, mixed model-based composite interval mapping (MCIM) was proposed by Zhu and Weir (1998). This method takes population phenotypic mean and various main genetic effects (additive effect, dominant effect, and epistatic effect) of QTL as fixed effects, and the markers, environment, markers environment interaction effect as random effects. QTL mapping analysis and effect value estimation were combined for joint QTL analysis in multiple environments, to improve the accuracy and efficiency of QTL mapping.

Research to uncover inheritance of soybean pubescence started about a century ago, three dominant mutants named *P1* (glabrous), *Ps* (sparse pubescence), and *Pd* (dense pubescence) were found related to PD (Owen, 1927; Bernard and Singh, 1969). Then these three genes were mapped on Chr01 (*Pd1*) (Cregan et al., 1999), Chr12 (*Ps*) (Specht et al., 2001; Bandillo et al., 2017), and Chr09 (*P1*) (Bandillo et al., 2017). With the rapid development of sequencing technology and data statistics, several QTLs related to pubescence length (PL) and PD were identified in the past decades. Two QTLs of PL (on Chr07 and Chr12) and two QTLs of PD (on Chr01 and Chr12) were identified using a recombinant inbred population (Oki et al., 2012). A major QTL on Chr12 and some other minor QTLs on Chr01, Chr02, Chr07, Chr08, Chr09, and Chr15 of PD were identified using a recombinant inbred line (RIL) population that derived from a cross between soybean cultivars *Kefeng 1* and *Nannong 1138-*2 (Du et al., 2009). Two and four QTLs related to PL and PD were mapped on Chr01, Chr12, and Chr01, Chr08, Chr12, and Chr20, respectively (Xing et al., 2013).

Genetic and molecular studies of the past years have shown that pubescence formation is regulated in a complex and precise way (Pattanaik et al., 2014). A large number of transcription factors (TFs) that regulate trichome development had been identified (Ishida et al., 2008). In recent years, availability of user-friendly genomic resources and easy-to-use bioinformatics tools, a number of studies have been conducted and some candidate genes identified with validation (Nakaya et al., 2020). The function of *GmCPR5* (ortholog of *Arabidopsis CPR5*) involved in pubescence development was tested by CRISPR/Cas9 (Campbell et al., 2019). Recently, genes responsible for the classic loci *Pd1*, *Ps*, and *P1* were cloned, and further analysis validated that these three genes can form a complex feedback network to precisely regulate pubescence formation in soybean (Liu et al., 2020).

With the well-established molecular technology and genetic transformation in soybean, genes responsible for pubescence and their function have been gradually clarified; however, there still exists limited knowledge on the molecular basis for pubescence development and regulatory pathways. Pubescence is controlled by major genes and polygenes; therefore, it is very difficult to identify these genes through conventional methods. Most populations used in previous studies were derived from cultivated parents with relatively narrow phenotypic differences, hence making it difficult to uncover the genetic information from wild soybean (Du et al., 2009).

In the present study, an inter-specific RIL population, which is derived from a cultivated soybean (*Glycine max* Merr.) (*Nannong 86-4*, *NN 86-4*) and a wild soybean line (*Glycine soja*) (*PI 342618B*), was used. The female parent *NN 86-4* has long and sparse pubescence, while the male parent *PI 342618B* has SD pubescence. The present study aimed to uncover the genetic architecture of PL and PD, to predict potential candidate genes, and to analyze the relationship between pubescence morphology and common cutworm (CCW; *Spodoptera litura* Fabricius) resistance.

## Materials and Methods

### Plant Material and Growth Conditions

The NJRINP contains 284 lines derived via single seed descent. All the 284 RILs along with their parents were planted in two environments viz. Jiangpu Experimental Station, Nanjing, Jiangsu Province (Latitude 33°03′ N; Longitude 118°63′ E) in 2011 (JP2011) and Baima Experimental Station, Nanjing, Jiangsu Province (Latitude 31°62′ N; Longitude 119°18′ E) in 2020 (BM2020). Each line was planted in one-row plot (length × width, 1.5 × 1 m). Field management followed standard conditions in each location.

### Phenotypic Analysis of Pubescence Length and Density

The third leaf from the top of each stem was taken from three plants in the field at V6 stage, then put in icebox and transported to the laboratory. Samples were dissected between the main vein and lateral vein near the base of the middle-leaflet of trifoliolate with 8 mm diameter puncher (avoiding primary veins) (Xing et al., 2013). Then the leaf discs were used to take photographs with an area of 12 mm^−2^ under a Leica stereo microscope. The software ImageJ^1^ was used to generate PL and PD. PD was converted from 12 mm^−2^ to an area of 10 mm^−2^ as the final density. As for PL, the pubescence on leaf surface was divided into two types: long and short, the length of three representative hairs of each type was measured, and average length was calculated by the weighted average method as

$$\bar{x}=\left({\bar{x}}_{l}\,f_{l}+{\bar{x}}_{s}\,f_{s}\right)/\left(f_{l}+f_{s}\right)$$

Where $\bar{x}$is a weighted average of PL, ${\bar{x}}_{l}$ is the average length of three representative long pubescence, *f*_*l*_ is the number of long pubescence, ${\bar{x}}_{s}$is the average length of three representative short pubescence, and *f*_*s*_ is the number of short pubescence.

### Statistical Analysis of Phenotypic Data

R software was used to draw the frequency distribution of phenotypic data. The descriptive statistics, such as mean, maximum and minimum, coefficient of variation (*CV*), correlation analysis, and ANOVA of traits were calculated using SAS software (SAS Institute, 2010. SAS/STAT software version 9.2. SAS Institute Inc., Cary, NC, United States). The broad-sense heritability (*h*^2^) for individual environments (Eq. 1) and combined environments (CE; Eq. 2) were computed following the formula proposed by Nyquist and Baker (1991).

(1) $$h^{2}=\sigma_{g}^{2}/\sigma_{p}^{2}$$

(2) $$h^{2}=\sigma_{g}^{2}/\left(\sigma_{g}^{2}+\sigma_{g\,e}^{2}/n+\sigma_{e}^{2}/n\,r\right)$$

Where $\sigma_{g}^{2}$ is the genotypic variance,$\sigma_{p}^{2}$ is the phenotypic variance (PV), $\sigma_{g\,e}^{2}$is the genotype by environment interaction variance, $\sigma_{e}^{2}$is the error variance, *n* is the number of environments, and *r* is the number of replications.

### Genetic Linkage Map Construction and Quantitative Trait Loci Mapping Analysis

In the present study, a high-density genetic linkage map was constructed using restriction site-associated DNA sequencing (RAD-seq) (Wang et al., 2016). Briefly, restriction enzymes were used to digest the purified genomic DNA firstly, then ligated digested products with P1 adapter by T4 DNA ligase. Every 24 RILs were collected together and randomly sheared ultrasonically and used a purification kit to purify DNA fragments. Next, the fragment end was repaired with a Quick Blunting kit (NEB). Finally, the collected fragments were enriched by PCR amplification and purified by a QIAquick PCR purification kit. In addition, standardized samples were sequenced on HiSeq 2000 instruments. The soybean genome sequence (*G. max*, Wm82.a1. v1) was used as a reference to predict digestion sites. A total of 5,728 bin markers were obtained from 89,680 single nucleotide polymorphisms, spanning a total genetic distance of 2,204.6 cM with an average distance of 0.4 cM between neighboring bins. The linkage map of bin markers was constructed for the RIL population using R with the package *LinkageMapView*.

Two QTL mapping models were adopted to map additive effect QTLs in the present study to discover the genetic basis of pubescence development. Firstly, CIM was implemented in WinQTLCart 2.5 software with a 10 cM window at a walking speed of 1 cM to map additive effect QTLs. The log of odd (LOD) threshold was determined by 1,000 permutation tests for each trait with an experimental-wise error rate of *P* = 0.05 to determine whether the QTL was significant. The QTLs detected with overlapping or closely linked confidence intervals (CIs) in different environments were recognized as the same QTL.

Secondly, QTL Network v2.0 software with MCIM model was used to map additive effect QTLs with the critical *F-*value calculated with 1,000 permutation tests. In addition, the QTL effects were estimated using the Markov Chain Monte Carlo (MCMC) method with 20,000 Gibbs sampler iterations. The significance level configuration of candidate interval selection, putative QTL detection, and QTL effects were calculated with an experiment-wise type I error under α = 0.05. The above analyses were done for individual environments (JP2011 and BM2020), averages from JP2011 and BM2020 were designated as the CE.

### Candidate Gene Prediction and Quantitative Real-Time PCR Analysis

The physical position of two flanking markers of major QTLs can be obtained by mapping sequencing data to Wm82.a1. v1. Both the model genes and annotation information within the physical genomic interval of major QTLs were obtained from SoyBase.^2^ The expression data of model genes were downloaded from SoyBase^3^ and Phytozome.^4^ The genes that expressed in young leaf were further classified according to Protein Analysis THrough Evolutionary Relationships (PANTHER).^5^ Based on functional annotations, PANTHER analysis, and available literatures, some genes were selected as candidate genes and their relative expression levels available on SoyBase and Phytozome were heatmapped using TBtools (Chen et al., 2020).

To perform qRT-PCR, total RNA of leaf samples of two parents (*NN 86-4* and *PI 342618B*) were isolated using RNA-prep Pure Plant Kit (TIANGEN DP-432, China) and full-length cDNA was reverse transcribed using a cDNA synthesis kit (Vazyme, R223) according to the protocol of the manufacturer. qRT-PCR was performed using ChamQ SYBR qPCR Master mix (Vazyme Q311) on Roche LightCycler 480 II. The housekeeping gene *GmActin11* was used as the reference. Three biological replicates were conducted for each analysis. The relative expressions of selected genes were computed using a 2^–△*CT*^ method (Livak and Schmittgen, 2001). The primer sequences for qRT-PCR are listed in Supplementary Table 1. The cluster analysis was performed using the Neighbor-Joining method in MEGA6 (Tamura et al., 2013). The percentage of replicate trees in which the associated taxa clustered together in the bootstrap test (1,000 replicates) are shown next to the branches.

### Resistance Evaluation to Common Cutworm

Common cutworm pupa stock was obtained from Soybean Research Institute of Nanjing Agriculture University. Third-instar larvae with uniform size were used for the experiment as described by Xing et al. (2017). The third leaf from the top of the stem of four kinds of pubescence morphology [long and sparse (LS); short and sparse (SS); long and dense (LD); and short and dense (SD)] was used to feed CCW larvae (five larvae per replication), and 10 different representative lines were selected for each pubescence morphology (PL/PD class). This experiment was conducted with three biological replicates per line. The initial larval weight was recorded and measured 3 days after forcibly feeding. The increased larval weight was subjected to ANOVA as a resistance indicator and multiple comparisons of different pubescence morphology (LS, SS, LD, and SD) were conducted via the least significant difference at 5% probability in SAS software.

## Results

### Phenotypic Analysis of Pubescence Length and Density

**FIGURE 1:**
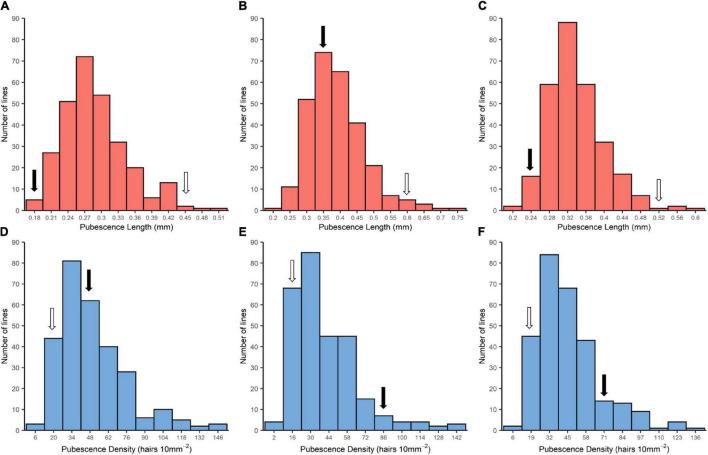
Frequency distribution of pubescence length (PL) and pubescence density (PD) of the RIL population NJRINP and parents. RIL, recombinant inbred line. **(A–C)** Frequency distribution of PL of JP2011, DT2020, and combined environment, respectively. **(D–F)** Frequency distribution of PD of JP2011, DT2020, and combined environment, respectively. The black arrow represents the wild soybean *PI 342618B* and the white arrow represents the cultivar *NN 86-4*.

PL and PD of 284 RILs and their parents in JP2011, BM2020, and CE are presented in Figure 1. PL and PD of the male parent *PI 342618B* were ranged 0.18–0.33 mm and 54.53–90.83 hairs 10 mm^−2^, respectively, which were shorter and denser than that of the female parent *NN 86-4* (0.45–0.58 mm and 17.22–21.67 hairs 10 mm^−2^) (Table 1 and Figure 1). The variation between two parents offered a broader genetic resource among the RILs for quantitative trait analysis. The mean value of some RILs exceeded two parents in both directions, indicating that RILs showed transgressive segregation in PL and PD (Figure 1). The phenotypic variation of PL and PD among RILs showed continuous distribution, suggesting both two traits are controlled by multiple genes, and thus suitable for QTL mapping.

**TABLE 1:** Descriptive statistics, broad-sense heritability for pubescence length and density in the RIL population NJRINP and parents.

Trait^a^	Env^b^	Parent^c^		RIL Population^d^					
		NN86-4	PI 342618B	Mean	Min	Max	SD	*CV* (%)	*h*^2^ (%)
PL	JP2011	0.45	0.18	0.29	0.14	0.71	0.06	17.1	81.7
	BM2020	0.58	0.33	0.39	0.17	0.90	0.09	14.1	87.6
	CE	0.51	0.25	0.34	0.20	0.69	0.06	15.4	54.6
PD	JP2011	17.22	54.53	48.51	5.74	181.77	26.23	26.4	91.4
	BM2020	21.67	90.83	38.90	3.95	163.33	25.10	23.4	94.9
	CE	19.44	72.68	44.82	10.31	129.60	21.50	25.4	65.7

*^a^PL = Pubescence length (mm) and PD = Pubescence density (10 mm^−2^).*

*^b^JP2011 = Jiangpu Experimental Station in 2011, BM2020 = Baima Experimental Station in 2020 & CE = Combined environment (average of JP2011 and BM2020).*

*^c^NN86-4 = Nannong86-4 (G. max) - female parent and PI 342618B = wild accession (G. soja) - male parent.*

*^d^Min, Max, SD, CV, and h^2^ represent minimum, maximum, standard deviation, error coefficient of variation and broad-sense heritability.*

In addition to the above, ANOVA and correlation analysis suggested that both traits (PL and PD) were influenced by environment and genotype by environment interaction (G × E) (Table 1, Supplementary Table 2, and Figure 1). The high *h*^2^ of the two traits (PL and PD) indicated that either of the traits is largely regulated by genetic factors. The correlation coefficients (*r*) of the same trait in different environments were ranged from 0.40 to 0.49, but a negative correlation was observed between PL and PD (*r* = −0.17 to −0.36; Supplementary Figure 1).

### Quantitative Trait Loci Mapping of Pubescence Length and Density by Composite Interval Mapping Method

**FIGURE 2:**
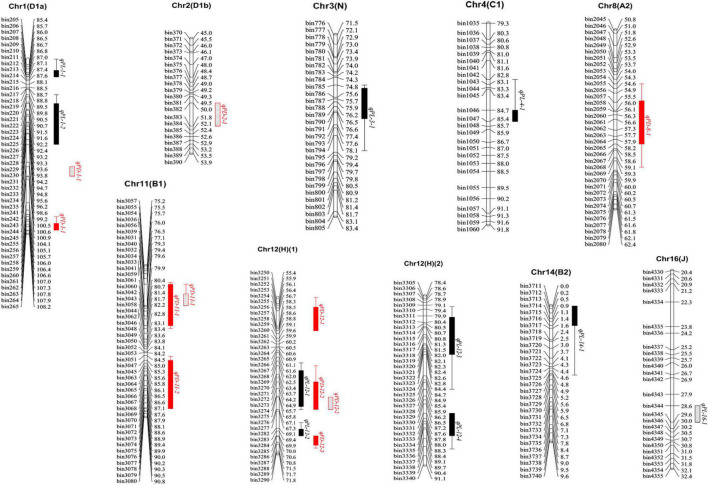
Locations of QTLs on genetic linkage map for pubescence length (PL) and pubescence density (PD). Black graphics represent the QTLs were responsible for PL. Red graphics represent the QTL were responsible for PD, and the filled graphics represent QTLs detected by the CIM method, while the hollow graphics represent QTLs detected by the MCIM method (Due to the high-density markers, this figure only shows the region where the QTL is located, the complete map is shown in Supplementary Figure 2). QTL, quantitative trait loci; CIM, composite interval mapping; MCIM, mixed model-based composite interval mapping.

The bin-marker distribution on each chromosome is shown in Supplementary Table 3 and Supplementary Figure 2. A total of 16 QTLs comprising nine and seven for PL and PD, respectively, were identified by the CIM model with LOD (3.4–14.0) and phenotypic variation explained (*R*^2^) (4.2%–15.8%) (Table 2). The highest number of seven QTLs (*qPL-12-1, qPL-12-2, qPL-12-3, qPL-12-4, qPD-12-1, qPD-12-2*, and *qPD-12-3*) was mapped on Chr12 followed by three QTLs (*qPL-1-1, qPL-1-2*, and *qPD-1-1*) on Chr01, two QTLs (*qPD-11-1, qPD-11-2*) on Chr11 (Table 2 and Figure 2). These results suggested that PL and PD are largely controlled by Chr12, Chr01, and Chr11.

**TABLE 2:** The QTLs identified for pubescence length and density in the inter-specific RIL population (NJRINP) with composite interval mapping (CIM) model.

QTL name^a^	Chr^b^	Pos (cM)^c^	LOD^d^	A^e^	*R*^2^ (%)^f^	CI (cM)^g^	Flanking markers	Physical interval	Env^h^
**Pubescence length (PL)**									
*qPL-1-1*	1	86.8	8.3	0.02	9.9	86.2–87.1	bin207-bin212	50906446–51270756	JP2011
*qPL-1-2*	1	93.2	6.4	0.02	7.7	90.7–93.8	bin221-bin231	51991887–52750166	JP2011
		95.6	5.3	0.02	6.8	93.8–96.2	bin230-bin235	52646512–53138307	PLCE
*qPL-3-1*	3	75.9	3.4	0.02	4.7	75.0–77.0	bin785-bin792	40733488–41489885	BM2020
*qPL-4-1*	4	84.8	3.5	0.01	4.2	84.5–85.4	bin1045-bin1048	46180297–46640424	PLCE
***qPL-12-1***	12	65.8	7.9	0.02	9.8	65–66.8	bin3273-bin3276	34739431–35072870	JP2011
		64.2	6.8	0.02	8.8	63.0–64.7	bin3269-bin3273	34404375–34792379	PLCE
***qPL-12-2***	12	69.4	7.0	0.02	8.8	69.2–69.9	bin3282-bin3285	35364334–35611487	JP2011
*qPL-12-3*	12	80.5	6.8	–0.02	8.2	80.0–80.8	bin3311-bin3315	37298939–37692157	JP2011
		82.0	4.6	–0.02	5.8	81.3–82.8	bin3315-bin3324	37587581–38228756	PLCE
*qPL-12-4*	12	87.7	4.3	–0.01	5.2	87.2–88.9	bin3331-bin3337	38605071–39044951	JP2011
*qPL-14-1*	14	0.1	3.4	0.02	4.6	0.0–1.4	bin3711-bin3716	1-926342	BM2020
**Pubescence density (PD)**									
*qPD-1-1*	1	107.4	8.6	–8.09	9.2	107.1–107.8	bin261-bin264	55426982–55905066	JP2011
		107.4	7.4	–7.57	9.1	106.9–107.8	bin260-bin264	55363092–55905066	BM2020
		107.4	11.5	–7.76	12.1	107.1–107.8	bin261-bin264	55426982–55905066	PDCE
*qPD-8-1*	8	56.3	4.4	5.60	4.5	55.4–58.2	bin2056-bin2066	13833592–14823585	JP2011
*qPD-11-1*	11	79.3	4.2	–5.72	5.0	77.1–81.4	bin3039-bin3043	25090171–29543061	BM2020
		79.5	6.7	–5.91	6.7	77.6–79.6	bin3031-bin3034	25192952–26800118	PDCE
*qPD-11-2*	11	88.9	4.1	–5.56	4.8	88.3–90.0	bin3071-bin3076	35896132–36898349	BM2020
		86.5	5.6	–5.44	5.7	85–87.1	bin3051-bin3068	32218337–35552114	PDCE
*qPD-12-1*	12	58.3	8.3	–8.23	9.8	56.3–58.8	bin3252-bin3259	32840286–33656092	JP2011
***qPD-12-2***	12	65.0	14.0	–10.43	15.8	64.2–67.1	bin3272-bin3277	34679960–35151243	JP2011
		66.8	14.0	–8.70	15.4	64.8–67.1	bin3272-bin3277	34679960–35151243	PDCE
***qPD-12-3***	12	70.0	11.4	–9.53	13.2	69.9–71.2	bin3284-bin3288	35489493–35857184	JP2011

*^a^Is designed by the traits (PL, pubescence length; PD, pubescence density), the chromosome that QTL is located on and the order of the QTL on the chromosome. The bold name represents the QTL with the same physical genomic position for PL and PD. The underlined name represents the QTL detected in both CIM and MCIM methods.*

*^b^Represents the chromosome that the QTL is located on.*

*^c^Means the genetic position on the chromosome of the QTL.*

*^d^Represents the log of odds (LOD) value at the peak likelihood of the QTL.*

*^e^Represents the estimated additive effect.*

*^f^Represents the phenotypic variance (%) explained by the QTL.*

*^g^Represents confidence interval.*

*^h^Represents the environmental condition. JP2011: Jiangpu Experimental Station in 2011, BM2020: Baima Experimental Station in 2020 & PLCE and PDCE: PL and PD of CE (combined environment, average of JP2011 and BM2020).*

*QTL, quantitative trait loci.*

For PL, nine QTLs were identified on five chromosomes (Chr01, Chr03, Chr04, Chr12, and Chr14) with phenotypic variation ranging from 4.2 to 9.9% and LOD ≥ 3.4 (Table 2 and Figure 2). Among these, QTLs *qPL-1-2*, *qPL-12-1*, and *qPL-12-3* were detected in JP2011 and CE with LOD values of 5.3–6.4, 6.8–7.9, and 4.6–6.8, *R*^2^ values of 6.8–7.7%, 8.8–9.8%, and 5.8–8.2%, respectively (Table 2). *qPL-1-1* was detected in JP2011 and accounted for an *R*^2^ value of 9.9%. The remaining five QTLs have relatively smaller *R*^2^ (4.2–8.8%). Aside from the additive effect of *qPL-12-3* and *qPL-12-4* that are negative, alleles for increased PL emanated from wild soybean *PI 342618B*, while others obtained increased PL additive effect from *NN 86-4* (Table 2).

The QTLs related to PD were identified on four chromosomes (Chr01, Chr08, Chr11, and Chr12) with LOD scores ranging from 4.1 to 14.0, which could explain phenotypic variation from 4.5 to 15.8% (Table 2 and Figure 2). Among them, *qPD-1-1* could be detected in both environments and CE with LOD_vs_*R*^2^ values of 7.4–11.5_vs_9.1%–12.1%, *qPD-11-1*, *qPD-11-2*, and *qPD-12-2* could be detected in one environment and CE with LOD_vs_*R*^2^ values of 4.2–6.7_vs_5.0–6.7%, 4.1–5.6_vs_4.8–5.7%, and 14.0_vs_15.4–15.8%, respectively. Besides, *qPD-12-3* was detected in only one environment (JP2011) but caused the highest *R*^2^ of 13.2%. The remaining two QTLs (*qPD-8-1* and *qPD-12-1*) were detected in single environment and could explain relatively lower phenotypic variation (Table 2). Interestingly, except *qPD-8-1* with a positive additive allele that increased PD allele from *NN 86-4*, all additive effects of other QTLs were negative with alleles for increasing PD from *PI 342618B*, suggesting PD is a domestication related trait which may have been lost or reduced during domestication. Hence the wild soybean *PI 342618B* contains beneficial alleles which could be exploited to increase PD in its domesticated progenies (Table 2).

By comparing QTLs for PL and PD, *qPL-12-1* and *qPD-12-2, qPL-12-2* and *qPD-12-3* overlapped, respectively. These four QTLs were flanked by markers *bin3269-bin3276, bin3272-bin3277, bin3282-bin3285*, and *bin3284-bin3288*, respectively (Table 2 and Figure 2). The increased pubescence traits alleles of PL and PD were from different parents, hence they could be the same QTL with pleiotropic effect, and explain the negative correlation between PL and PD.

### Additive Quantitative Trait Loci Conferring Pubescence Length and Density Detected by Mixed Model-Based Composite Interval Mapping Method

In all, one additive QTL (*qPL-16-1*) of PL and four additive QTLs (*qPD-1-2, qPD-2-1, qPD-11-1*, and *qPD-12-2*) of PD were detected by MCIM implemented in QTL Network v2.0 software.

For PL, *qPL-16-1* could cause phenotypic variation of 3.0%. It is a novel locus detected for the first time with an increased PL additive effect from *NN 86-4* (Table 3). A total of four QTLs were detected related to PD accounted for 0.8%–12.8% phenotypic variation, among which *qPD-12-2* could explain the phenotypic variation of 12.8%. All the QTLs inherited their increased PD alleles from *PI 342618B* (Table 3), supporting our earlier assertion that pubescence may be one of the domestication syndrome traits in soybean. *qPL-12-1* and *qPD-12-2* by CIM were overlapped with *qPD-12-2* by MCIM (Tables 2, 3), hence this locus was considered as the major QTL for pubescence development in the present panel. In addition, *qPD-11-1* was detected by CIM and MCIM, respectively (Tables 2, 3). Therefore this region was considered as major QTL for regulating PD in this population.

**TABLE 3:** The additive QTLs identified for pubescence length and density in the inter-specific RIL population (NJRINP) with the mixed model-based composite interval mapping (MCIM) method.

QTL name^a^	Chr^b^	Pos (cM)^c^	A^d^	*p*-value	*R*^2^ (%)^e^	CI (cM)^f^	Flanking markers	Physical interval
**Pubescence length (PL)**								
*qPL-16-1*	16	29.6	0.01	0.000027	3.0	28.6–29.6	bin4344-bin4345	4896662–5068829
**Pubescence density (PD)**								
*qPD-1-2*	1	100.2	–6.62	0.000000	6.4	99.2–100.5	bin242-bin244	53446303–53888306
*qPD-2-1*	2	51	–3.53	0.000267	0.8	50.0–51.8	bin382-bin383	11692635–12078676
*qPD-11-1*	11	79.1	–5.30	0.000000	4.2	78.1–79.3	bin3031-bin3032	25192952–26661690
***qPD-12-2***	12	66.8	–8.90	0.000000	12.8	65.8–67.1	bin3274-bin3277	34792380–35151243

*^a^Is designed by the traits (PL: pubescence length; PD: pubescence density), the chromosome that QTL is located on and the order of the QTL on the chromosome. The underlined name represents the QTL detected in both CIM and MCIM methods. QTL, quantitative trait loci.*

*^b^Represents the chromosome that the QTL is located on.*

*^c^Means the genetic position on the chromosome of the QTL.*

*^d^Represents the estimated additive effect.*

*^e^Represents the phenotypic variance (%) explained by the QTL.*

*^f^Represents confidence interval.*

### Candidate Gene Screening of Pubescence Length and Density Within Major Quantitative Trait Loci

For PL: *qPL-1-2, qPL-12-1*, and *qPL-12-3* were detected in JP2011 and CE, which were considered as major QTLs. These three QTLs contain 131, 60, and 80 genes (total 271), respectively (Wm82.a1. v1). For PD, *qPD-1-1* was mapped in both JP2011 and BM2020 and CE, while *qPD-11-1* and *qPD-12-2* were detected by both CIM and MCIM. Besides, *qPD-12-3* with the LOD value of greater than 10 caused 13.2% phenotypic variation in PD and overlapped with *Pubescence density 2-7* (Du et al., 2009), hence this could be considered as a major QTL. *qPD-11-2* was not considered as a major QTL due to lower phenotypic variation. Within the genomic regions of *qPD-1-1*, *qPD-11-1*, *qPD-12-2*, and *qPD-12-3*, 70, 75, 41, and 39 model genes (total 225) were downloaded, respectively.

A total 247 of 271 genes for PL and 200 of 225 genes for PD were informatively annotated, respectively (Supplementary Tables 4, 5). Expression data of distinct tissues in soybean have been completed in previous research (Severin et al., 2010). A total 203 and 151 genes of PL and PD with expression in young leaf were selected for PANTHER analysis (Wm82.a2. v1) (Supplementary Tables 6, 7). For PL, 93 out of the 203 genes were included in PANTHER protein classes and involved in 14 pathways. Then 17 out of the 93 genes were considered as the candidate genes according to literatures and annotation information (Table 4). Furthermore, there were other four candidate genes that did not include in protein classes (Table 4). For PD, 70 out of the 151 genes were included in PANTHER protein classes and involved in nine pathways. A total of nine genes were considered as the candidate genes according to literatures and annotation information. Except for the above genes, three candidate genes were not included in protein classes (Table 4). Among above 21 and 12 candidate genes of PL and PD, respectively, *Glyma.12g185500*, *Glyma.12g188600*, and *Glyma.12g188800* were responsible for both PL and PD, suggesting a possible pleiotropic effect of some candidate genes.

**TABLE 4:** Candidate genes within major QTL regions identified based on gene annotation, PANTHER analysis, and available literatures.

Gene names	Gene annotation	PANTHER protein class	References	QTL
*Glyma.01g194600*	SANT/MYB DOMAIN	Chromatin/chromatin-binding, or -regulatory protein (PC00077)	Hua et al., 2021; Yuan et al., 2021	*qPL-1-2*
*Glyma.12g184700*	MYB FAMILY TRANSCRIPTION FACTOR-RELATED	Gene-specific transcriptional regulator (PC00264)		*qPL-12-1*
*Glyma.12g193300*	MYB-LIKE DNA-BINDING PROTEIN MYB	*		*qPD-12-3*
***Glyma.12g195200***	MYB-LIKE DNA-BINDING PROTEIN MYB	Gene-specific transcriptional regulator (PC00264)		*qPD-12-3*

*Glyma.01g197900*	Myc-TYPE, BASIC HELIX-LOOP-HELIX (bHLH) DOMAIN	Gene-specific transcriptional regulator (PC00264)	Hülskamp et al., 1994; Schellmann et al., 2002; Esch et al., 2003; Ishida et al., 2008; Xu et al., 2018	*qPL-1-2*
*Glyma.01g198000*	TRANSCRIPTION FACTOR BHLH18-RELATED	Gene-specific transcriptional regulator (PC00264)		*qPL-1-2*
***Glyma.01g198100***	BASIC HELIX-LOOP-HELIX (bHLH) DOMAIN	Gene-specific transcriptional regulator (PC00264)		*qPL-1-2*
*Glyma.12g188600*	BASIC HELIX-LOOP-HELIX (bHLH) DOMAIN	Gene-specific transcriptional regulator (PC00264)		*qPL-12-1, qPD-12-2*
*Glyma.12g214900*	Myc-TYPE, BASIC HELIX-LOOP-HELIX (bHLH) DOMAIN	Gene-specific transcriptional regulator (PC00264)		*qPL-12-3*
*Glyma.12g216800*	TRANSCRIPTION FACTOR BHLH123	Gene-specific transcriptional regulator (PC00264)		*qPL-12-3*
*Glyma.12g196700*	Myc-TYPE, BASIC HELIX-LOOP-HELIX (bHLH) DOMAIN	*		*qPD-12-3*
*Glyma.12g196900*	Myc-TYPE, BASIC HELIX-LOOP-HELIX (bHLH) DOMAIN	Gene-specific transcriptional regulator (PC00264)		*qPD-12-3*

*Glyma.12g182700*	WD40-REPEAT-CONTAINING DOMAIN	*	Ishida et al., 2008; Liu et al., 2020	*qPL-12-1*
*Glyma.12g183500*	WD40-REPEAT-CONTAINING DOMAIN	*		*qPL-12-1*
***Glyma.12G187200 (Ps)***	WD40-REPEAT-CONTAINING DOMAIN	*		*qPL-12-1, qPD-12-2*
*Glyma.12g214400*	WD40-REPEAT-CONTAINING DOMAIN	Chromatin/chromatin-binding, or -regulatory protein (PC00077)		*qPL-12-3*
*Glyma.12g214600*	WD40-REPEAT-CONTAINING DOMAIN	*		*qPL-12-3*

*Glyma.12g183700*	VPS4 OLIGOMERISATION, C-TERMINAL	Cytoskeletal protein (PC00085)	Kang et al., 2016; Chang et al., 2019; Li et al., 2019; Tang et al., 2020	*qPL-12-1*
*Glyma.12g185500*	RHO GTPASE-ACTIVATING PROTEIN 3-RELATED	Cytoskeletal protein (PC00085)		*qPL-12-1, qPD-12-2*
*Glyma.12g215200*	MICROTUBULE MOTOR ACTIVITY	Cytoskeletal protein (PC00085)		*qPL-12-3*
*Glyma.12g219400*	MYOSIN	Cytoskeletal protein (PC00085)		*qPL-12-3*
*Glyma.01g244400*	GAMMA TUBULIN COMPLEX PROTEIN	Cytoskeletal protein (PC00085)		*qPD-1-1*
*Glyma.11g175500*	NUDC DOMAIN-CONTAINING PROTEIN 2	Cytoskeletal protein (PC00085)		*qPD-11-1*

*Glyma.12g215700*	POLY(ADP-RIBOSE) POLYMERASE, CATALYTIC DOMAIN	Gene-specific transcriptional regulator (PC00264)	Gan et al., 2007; Zhou et al., 2011; Sun et al., 2015	*qPL-12-3*

*Glyma.01g240100* (*Pd1*)	HOMEOBOX-LEUCINE ZIPPER PROTEIN MERISTEM L1-RELATED	Gene-specific transcriptional regulator (PC00264)	Zhang et al., 2012	*qPD-1-1*
*Glyma.12g188800*	HOMEOBOX PROTEIN TRANSCRIPTION FACTORS	Gene-specific transcriptional regulator (PC00264)		*qPL-12-1, qPD-12-2*
***Glyma.12g194400***	HOMEOBOX DOMAIN	Gene-specific transcriptional regulator (PC00264)		*qPD-12-3*

***Glyma.01g195900***	AP2/ERF DOMAIN	Gene-specific transcriptional regulator (PC00264)	Tan et al., 2015; Sun et al., 2017; Shang et al., 2020	*qPL-1-2*
*Glyma.01g206700*	AP2 DOMAIN	Gene-specific transcriptional regulator (PC00264)		*qPL-1-2*

***Glyma.12g195900***	CYCLIN	*	Meijer and Murray, 2001; Gao et al., 2017	*qPD-12-3*

*Glyma.12g219700*	FAMILY NOT NAMED	*	Pu et al., 2008	*qPL-12-3*

*Indicates these genes are selected based on gene annotation and literatures. The gene name in bold font represents the important candidate gene.

*QTL, quantitative trait loci; PANTHER, Protein Analysis THrough Evolutionary Relationships.*

**FIGURE 3:**
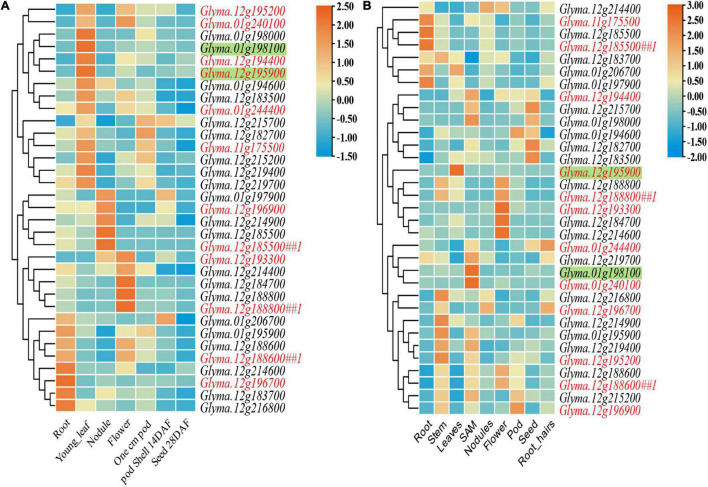
Heatmap of expression data from SoyBase **(A)** and Phytozome **(B)** for 21 and 12 candidate genes of pubescence length (PL) and pubescence density (PD). The candidate gene names of PL and PD are in black and red, respectively. The gene name with *##1* represents the candidate gene for both PL and PD. The gene name with shadow represents the important candidate genes identified by differential expression in two parents.

**FIGURE 4:**
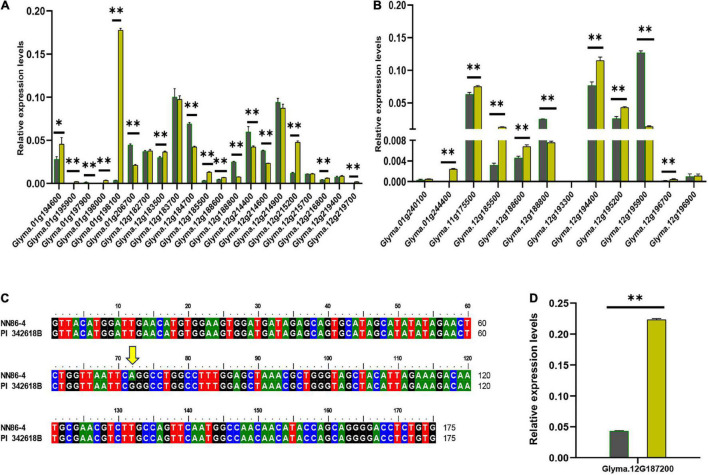
Difference analysis of candidate genes of pubescence length (PL) and pubescence density (PD). **(A,B)** The expression levels of 21 and 12 candidate genes of PL **(A)** and PD **(B)**, respectively, in parents *PI 342618B* (black column) and *NN 86-4* (yellow column). **(C)** DNA sequence alignment of the seventh exon of *Glyma.01g240100* (*Pd1*) in two parents. The yellow arrow represents the SNP between two parents. **(D)** Expression analysis of *Glyma.12G187200* (*Ps*) in parents *PI 342618B* (black column) and *NN 86-4* (yellow column) (*P ≤ 0.05; ^**^P ≤ 0.01).

Most of these 30 (three genes are the same for both PL and PD) candidate genes with relative higher expression in young leaf or shoot apical meristem (SAM) (Supplementary Table 8 and Figure 3). Their expression was measured subsequently by qRT-PCR in the leaves of two parents: *PI 342618B* and *NN 86-4* (Figures 4A,B). Eight and five genes of PL and PD, respectively, were expressed differentially by more than 2-fold between two parents. For PL, *Glyma.01g198100* expressed more than 50 folds higher in *NN 86-4* than *PI 342618B* (Figure 4A). For PD, *Glyma.12g195900* has an expression level of more than 9-fold in *PI 342618B* compared with *NN 86-4* (Figure 4B). Also, *Glyma.12g195900* is the homologous gene of *CYCU1* which could promote meristem cell division in *Arabidopsis* (Peng et al., 2014). Therefore, *Glyma.01g198100* and *Glyma.12g195900* were considered as the important candidate genes for PL and PD, respectively.

*Glyma.01g240100*, one of the 80 model genes of *qPD-1-1*, has been identified as *Pd1* due to a T to C single nucleotide polymorphism (SNP) in the last exon (Liu et al., 2020). Only one G to A synonymous mutation was detected in the seventh exon between *PI 342618B* and *NN 86-4* in the present study (Figure 4C and Supplementary Figure 3). *Glyma.12G187200*, a candidate gene of major QTL *qPL-12-1* and *qPD-12-2* with incompletely annotated information, was identified as *Ps* due to different copy numbers and expression level (Liu et al., 2020). The expression level of *Glyma.12G187200* in the leaves of *NN 86-4* was fivefold greater than in *PI 342618B* (Figure 4D). These results suggested that *Glyma.01g240100* may not be the candidate gene of *qPD-1-1*, and *Glyma.12G187200* was the candidate gene that not only controls PD of soybean leaves, but it also contributes to PL in the present study. Then a cluster analysis of the above candidate genes was conducted with their homologs, which have been known to be associated with pubescence development in several species (Supplementary Figure 4). Most candidate genes clustered together with their homologs, with seven were reliable (bootstrap values > 65%), such as *Ps* and *Pd1* (Supplementary Figure 4). Among them, *Glyma.01g195900* and *Glyma.12G187200* (*Ps*) of PL and *Glyma.12g194400*, *Glyma.12G187200* (*Ps*), *Glyma.12g195200*, and *Glyma.12g195900* of PD were suggested as important candidate genes due to significant differences in the expression between parents. Based on qRT-PCR and cluster analysis, three (*Glyma.01g195900*, *Glyma.01g198100*, and *Glyma.12G187200*) and four (*Glyma.12G187200*, *Glyma.12g194400*, *Glyma.12g195200*, and *Glyma.12g195900*) important candidate genes were identified for PL and PD, respectively.

### Resistance of Different Pubescence Morphology Lines to Common Cutworm

**FIGURE 5:**
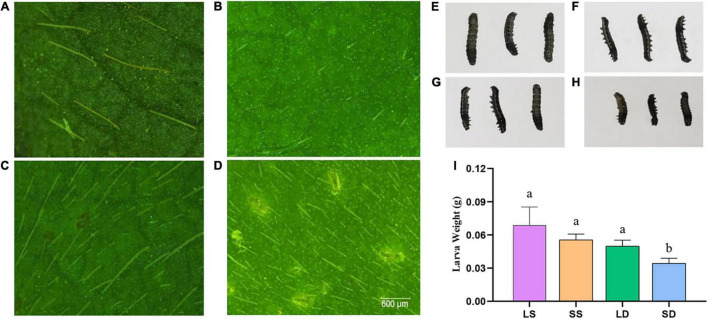
The resistance to CCW of lines with different pubescence length and density. **(A–D)** Typical photographs of four different pubescence morphology. **(A)** Long and sparse (LS); **(B)** short and sparse (SS); **(C)** long and dense (LD); **(D)** short and dense (SD). CCW, common cutworm. **(E–H)** Larvae morphology after feeding with leaves of four different pubescence morphology. (**E–H** corresponds to **A–D**, respectively). **(I)** The added larval weight was inoculated with the leaves of four different pubescence morphology. Significant differences were shown by different letters (*P* ≤ 0.05).

To determine the relationship between pubescence morphology and resistance to CCW, an antibiotic test was carried out using lines with four types of pubescence morphology and the typical photographs of pubescence are shown in Figures 5A–D. There was a significant difference between increased larval weight of feeding with leaves with SD pubescence (similar to the pubescence morphology of wild soybean *PI 342618B*) and leaves with the other three pubescence morphology types. The former (SD, viz. wild soybean pubescence morphology) had the strongest resistance to CCW, followed by LD, SS, and LS (Figures 5E–I). The increased larval weight had positive and negative correlations with PL and PD, respectively (Table 5).

**TABLE 5:** The correlation analysis of pubescence traits and resistance to CCW in four kinds of different pubescence morphology lines.

Traits	PL2011	PL2020	PD2011	PD2020	PLCE	PDCE
PL2020	0.69**					
PD2011	–0.50**	–0.21				
PD2020	–0.40**	–0.25	0.69**			
PLCE	0.90**	0.94**	–0.37**	–0.35**		
PDCE	–0.49**	–0.25	0.93**	0.91**	–0.39**	
LW	0.51**	0.43**	–0.31**	–0.30	0.50**	–0.33**

***PL2011**, pubescence length (PL) in Jiangpu Experimental Station in 2011; **PL2020**, PL in Baima Experimental Station in 2020; **PLCE**, PL of the combined environment (average of JP2011 and BM2020).*

***PD2011**, pubescence density (PD) in Jiangpu Experimental Station in 2011; **PD2020**, PD in Baima Experimental Station in 2020; **PDCE**, PD of the combined environment (average of JP2011 and BM2020).*

***LW**, increased larval weights of CCW. CCW, common cutworm.*

(**P ≤ 0.01).

## Discussion

### Genetic Basis of Pubescence Length and Density

Pubescence can form a physical barrier that protects plants against various biotic and abiotic stresses (Kennedy, 2003; Kang et al., 2016). The effectiveness of this preventive mechanism depends on the length and density of pubescence (Hua et al., 2021). Therefore, it is necessary to understand the genetic mechanisms associated with polygenic quantitative characters PL and PD. Although several QTLs related to PL and PD have been identified and reported over the past decades, most of them have large interval regions due to the small mapping population (<200 lines) and low-density genetic map based on simple sequence repeat (SSR) markers (Du et al., 2009; Oki et al., 2012). These may be difficult to be used in practical plant breeding and predicting probable candidate genes.

In the present study, two QTL mapping methods were used to complement and validate the results of each other and improve the accuracy of QTL mapping. A total of nine and seven QTLs were detected of PL and PD by CIM, respectively, while one and four QTLs were mapped by MCIM. By comparing the results of two methods, two QTLs (*qPD-11-1* and *qPD-12-2*) related to PD were detected in both methods, and *qPD-12-2* (Chr12) was the same as previously reported QTLs viz. *Pubescence density 2-8, Pubescence density 3-2*, and *PD12-1* (Du et al., 2009; Oki et al., 2012; Xing et al., 2013), suggesting these loci play a role in pubescence development. Besides, the CIM method detected more QTLs with higher additive effect and *R*^2^ value while the MCIM method mapped QTLs with narrow CIs. It was possible to miss some important loci if only the MCIM method was used, thus, it is better to use two methods.

By comparing QTLs detected in two methods with the reported ones in previous studies, *qPL-12-2* (Chr12) with an interval of 35,364,334–35,611,487 bp overlapped with *Pubescence length 1-2*, which had a larger CI (35,108,089–36,780,375 bp) (Oki et al., 2012). *qPL-12-3* (Chr12) was overlapped with *PL12-1* (Xing et al., 2013). The remaining eight QTLs related to PL were detected for the first time in the present study (Tables 2, 3 and Figure 2). For PD, a total of nine QTLs were detected by CIM and MCIM. In addition to *qPD-12-2* mentioned in the previous paragraph, *qPD-12-3* (Chr12) was overlapped with *Pubescence density 2-7* (Du et al., 2009); *qPD-1-1* (Chr01) and *qPD-2-1* (Chr02) were same to *Pubescence density 3-1*, *PD1-1* (Oki et al., 2012; Xing et al., 2013) and *Pubescence density 2-5* (Du et al., 2009), respectively, but in a narrower genomic region in the present study. The remaining five QTLs were detected for the first time. Most QTLs were novel indicating the distinct and abundant genetic architecture of pubescence in wild soybean. It also suggests the need to utilize more diverse parents to develop a mapping population to reveal the complex genetic basis of pubescence development in soybean and provide more valuable information for the gene identification related to pubescence development. In addition, the majority of QTLs identified in the present study were in small physical genomic regions, suggesting the importance of using a high-resolution genetic map for QTL detection and candidate gene exploration.

Although many studies had demonstrated that dense and long pubescence have higher resistance to abiotic stress (Turnipseed, 1977; Gunasinghe et al., 1988), the purpose of soybean breeding is not always to increase density and length of pubescence. It is important to keep PL and PD within a suitable range for the better growth and development of plants. In addition, it was found that there was a negative correlation between PL and PD in the present study, hence, materials with dense and long pubescence may not be easy to obtain. Our results demonstrated that soybean leaves with SD viz. wild soybean pubescence morphology instead of LS viz. cultivar soybean pubescence morphology had the stronger resistance to CCW thus the former can be used in soybean breeding.

### Candidate Gene Analysis of Pubescence Length and Density

It is of great significance for both theoretical research and breeding practice to identify the candidate genes of major QTL regions of pubescence traits in soybean. Many factors were identified to be related to trichome development in other species, providing the useful information to explore candidate genes of soybean pubescence development. The mechanism of *Arabidopsis* trichome development has been comprehensively explained (Shang et al., 2020). The core regulatory components are the R2R3-MYB/basic helix-loop-helix (bHLH)/WD complex (Ishida et al., 2008). R3-MYB negatively regulates trichome formation by competing with R2R3-MYB for binding to bHLH (Hülskamp et al., 1994; Schellmann et al., 2002; Esch et al., 2003). In tomato, bHLH TF (*SlMYC1*) and R2R3-MYB TFs (*SlTHM1* and *SlMYB52*) play an important role in the formation of trichomes (Xu et al., 2018; Yuan et al., 2021). The fiber initiation and elongation are somehow similar to trichome development. In cotton, R2R3-MYB TF *GhMYB109* is required for cotton fiber development (Pu et al., 2008) and the homologues of *GhMYB109* in tomatoes might also participate in the regulation of trichome elongation (Hua et al., 2021). Therefore, MYB TF, bHLH TF, and WD play an extremely important role in both unicellular and multicellular trichome development.

Recently, actin filaments and microtubules were reported to play coordinated but distinct roles in the formation of tomato trichome (Chang et al., 2019). Both in *Arabidopsis* and tomatoes, mutations in genes of SCAR/WAVE complex could lead to distorted trichomes (Kang et al., 2016; Chang et al., 2019; Li et al., 2019). In soybean, *GmNAP1* was involved in actin filament assembling during trichome and pavement cell development (Campbell et al., 2016; Tang et al., 2020). Thus, actin and microtubules were identified as having an undeniable role in trichome development in recent years.

Additionally, a set of C2H2 zinc finger TFs, such as *GIS*, *GIS2*, *GIS3*, *ZFP5*, and *Hair* (*H*) gene, were detected to be involved in trichome development in *Arabidopsis* and tomatoes, respectively (Gan et al., 2007; Zhou et al., 2011; Sun et al., 2015; Chang et al., 2018). AP2 and AP2/ERF TFs: *TAR1* and Hairy Leaf 6 (*HL6*), play an important role in trichome development in *A. annua* (Tan et al., 2015) and rice (Sun et al., 2017; Shang et al., 2020). Besides, the WUS-type homeobox gene *OsWOX3B* was found to be required for macro-hair initiation and trichome development in rice (Zhang et al., 2012). Cyclins were involved in the transition of the cell cycle and function as positive regulators of cell proliferation in eukaryotes (Meijer and Murray, 2001) and a B-type cyclin gene, *SlCycB2*, plays key roles in trichome initiation in tomatoes (Gao et al., 2017). These results suggested that the above factors may be functional during trichome development.

A total of 22 and 13 candidate genes (together with *Ps*) were identified for PL and PD, respectively, based on PANTHER analysis, expression data, and literatures in the present study (Table 4). It should be noted that genes within the physical genomic interval that are not annotated and/or have no expression in young leaves may be ignored. A cluster analysis was conducted of these candidate genes and the homologs mentioned above (Supplementary Figure 4). The genes *Ps* and *Pd1*, which have known to be related to soybean *PD*, were clustered with homologous genes of other species, indicating the reliability of this analysis method. However, more study is needed for their functional validation.

## Conclusion

A total of 10 and 9 QTLs of PL and PD were detected, respectively, from which three and four important candidate genes were identified. PL negatively correlated with PD and leaves with short and dense pubescence viz. wild soybean pubescence morphology had the highest resistance to CCW.

## Data Availability Statement

The original contributions presented in the study are included in the article/Supplementary Material, further inquiries can be directed to the corresponding author/s.

## Author Contributions

TZ and GX conceived and designed the experiments. YL, LC, XL, NZ, YX, YW, ZL, LT, and HY performed the experiments. YL and FC analyzed the data. YL drafted the manuscript. GX, TZ, and BK revised the manuscript. All authors contributed to the article and approved the submitted version.

## Conflict of Interest

The authors declare that the research was conducted in the absence of any commercial or financial relationships that could be construed as a potential conflict of interest.

## Publisher’s Note

All claims expressed in this article are solely those of the authors and do not necessarily represent those of their affiliated organizations, or those of the publisher, the editors and the reviewers. Any product that may be evaluated in this article, or claim that may be made by its manufacturer, is not guaranteed or endorsed by the publisher.

## Footnotes

1
https://imagej.en.softonic.com/

2
https://www.soybase.org/genomeannotation/

3
https://www.soybase.org/soyseq/

4
https://genome.jgi.doe.gov/portal/pages/dynamicOrganismDownload.jsf?organism=Phytozome

5
http://www.pantherdb.org/

## Supplementary Material

Supplementary Figure 1 Pearson’s correlation analysis of pubescence length (PL) and pubescence density (PD) (^**^*P* ≤ 0.01). PL2011, the PL in Jiangpu experimental station in 2011; PL2020, the PL in Baima experimental station in 2020; PLCE, the PL of combined environment (the average of JP2011 and BM2020). PD2011, the PD in Jiangpu experimental station in 2011; PD2020, the PD in Baima experimental station in 2020; PDCE, the PD of combined environment (the average of JP2011 and BM2020).

Supplementary Figure 2 High-density genetic linkage map of 20 chromosomes in the NJRINP constructed based on the RAD-Seq. The different colors represent the distinct marker density.

Supplementary Figure 3 DNA sequence alignment of the last exon of *Glyma.01g240100 (Pd1)* in two parents.

Supplementary Figure 4 Phylogenetic relationship between predicted candidate genes and their homologs based on literature. AT, *Arabidopsis thaliana* (thale cress); Glyma, *Glycine max* (Linn.) Merr. (soybean); Os, *Oryza sativa* (rice); Sl, *Solanum lycopersicum* (tomato); Am, *Antirrhinum majus* L. (Snapdragon). Gene names from different species are shown by different colors. Gene names with stars behind them indicate genes that can cluster with homologous genes.
