# Supplementary figures and images for: Genetic Architecture and Candidate Genes for Pubescence Length and Density and Its Relationship With Resistance to Common Cutworm in Soybean
Source: Front Plant Sci. 2022 Jan 7;12:771850. doi: 10.3389/fpls.2021.771850 (PMC8776989; doi:10.3389/fpls.2021.771850)

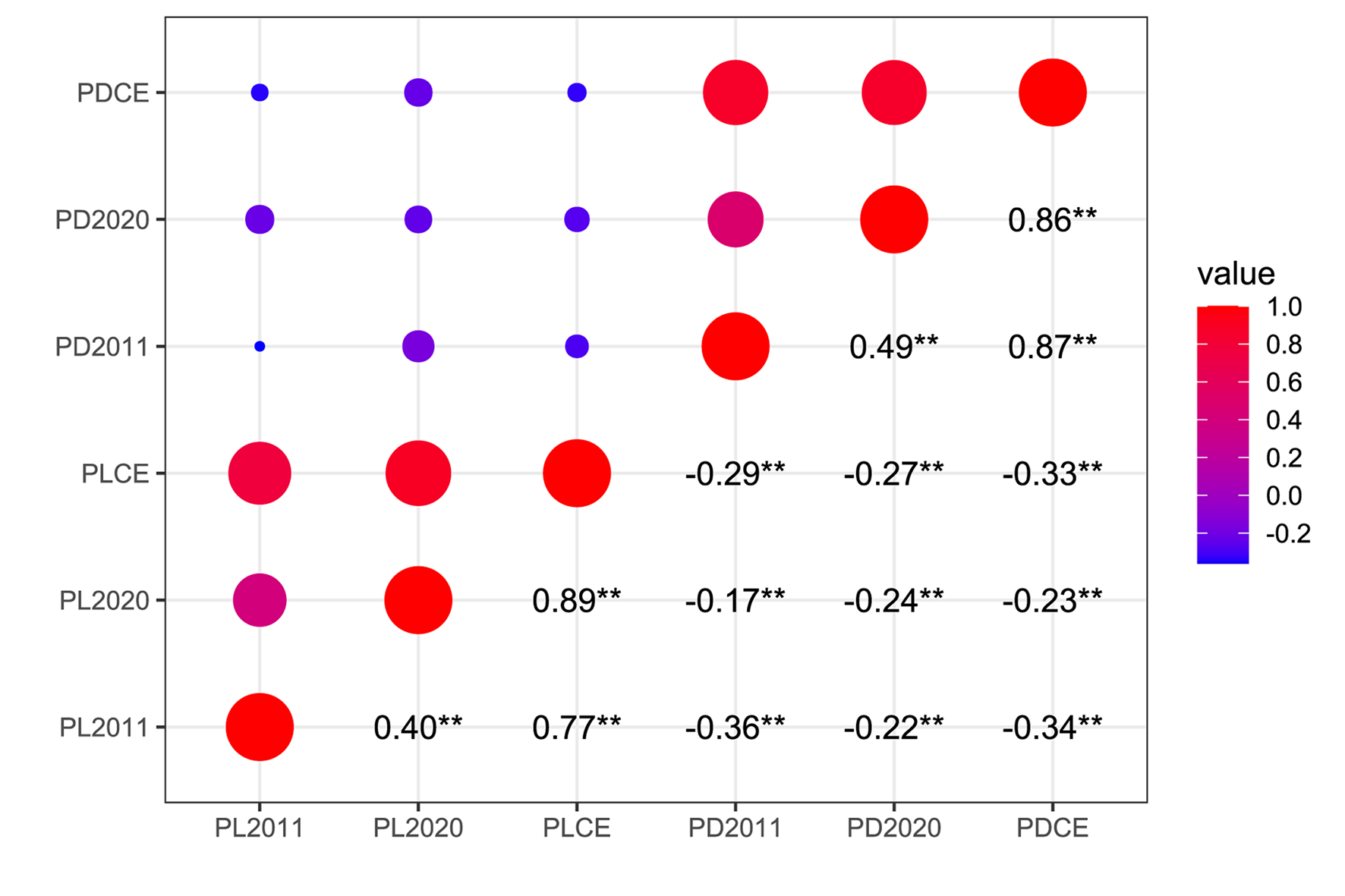

Supplement: Supplementary Figure 1 — Pearson’s correlation analysis of pubescence length (PL) and pubescence density (PD) (**P ≤ 0.01). PL2011, the PL in Jiangpu experimental station in 2011; PL2020, the PL in Baima experimental station in 2020; PLCE, the PL of combined environment (the average of JP2011 and BM2020). PD2011, the PD in Jiangpu experimental station in 2011; PD2020, the PD in Baima experimental station in 2020; PDCE, the PD of combined environment (the average of JP2011 and BM2020). [file Image_1.tif]

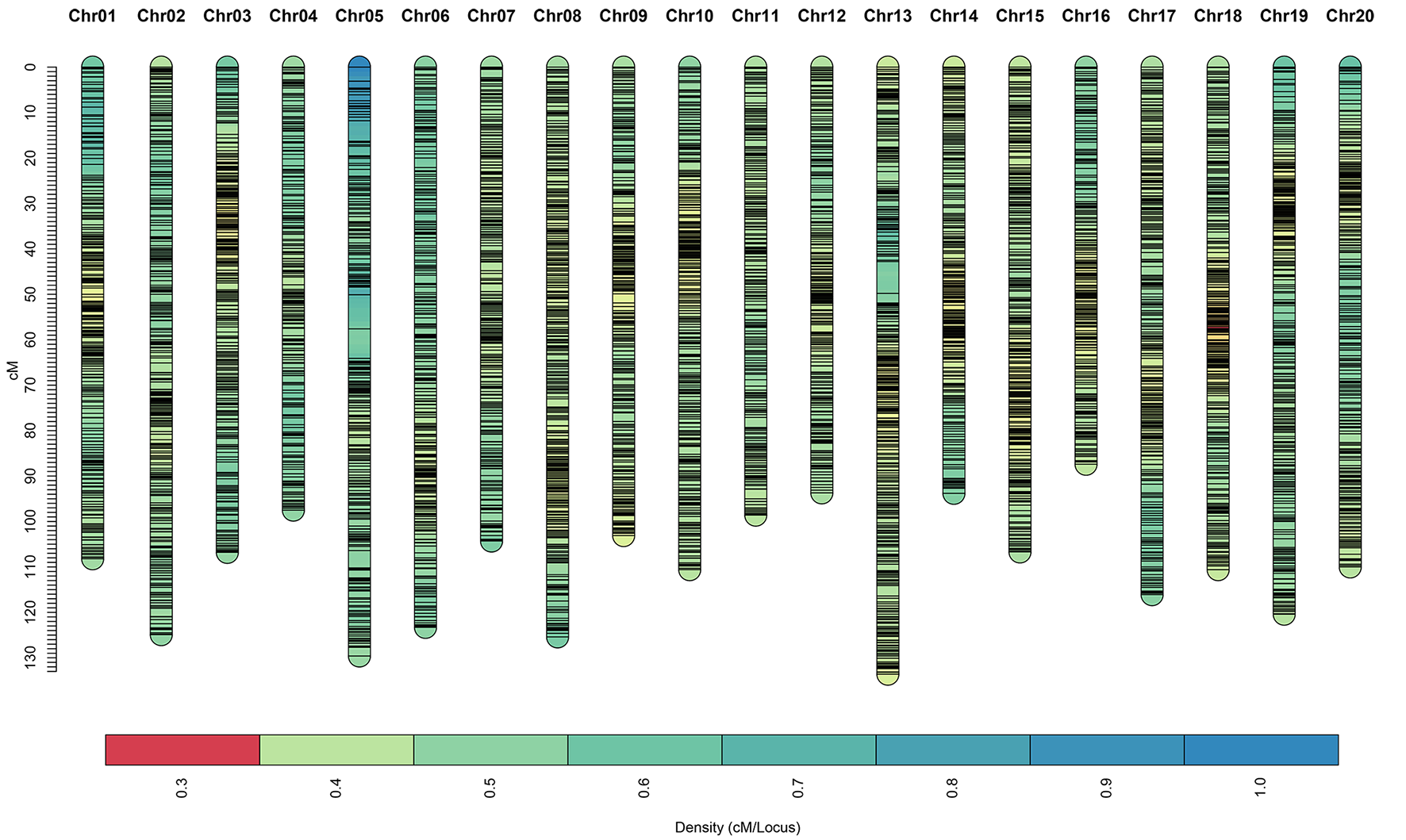

Supplement: Supplementary Figure 2 — High-density genetic linkage map of 20 chromosomes in the NJRINP constructed based on the RAD-Seq. The different colors represent the distinct marker density. [file Image_2.tif]

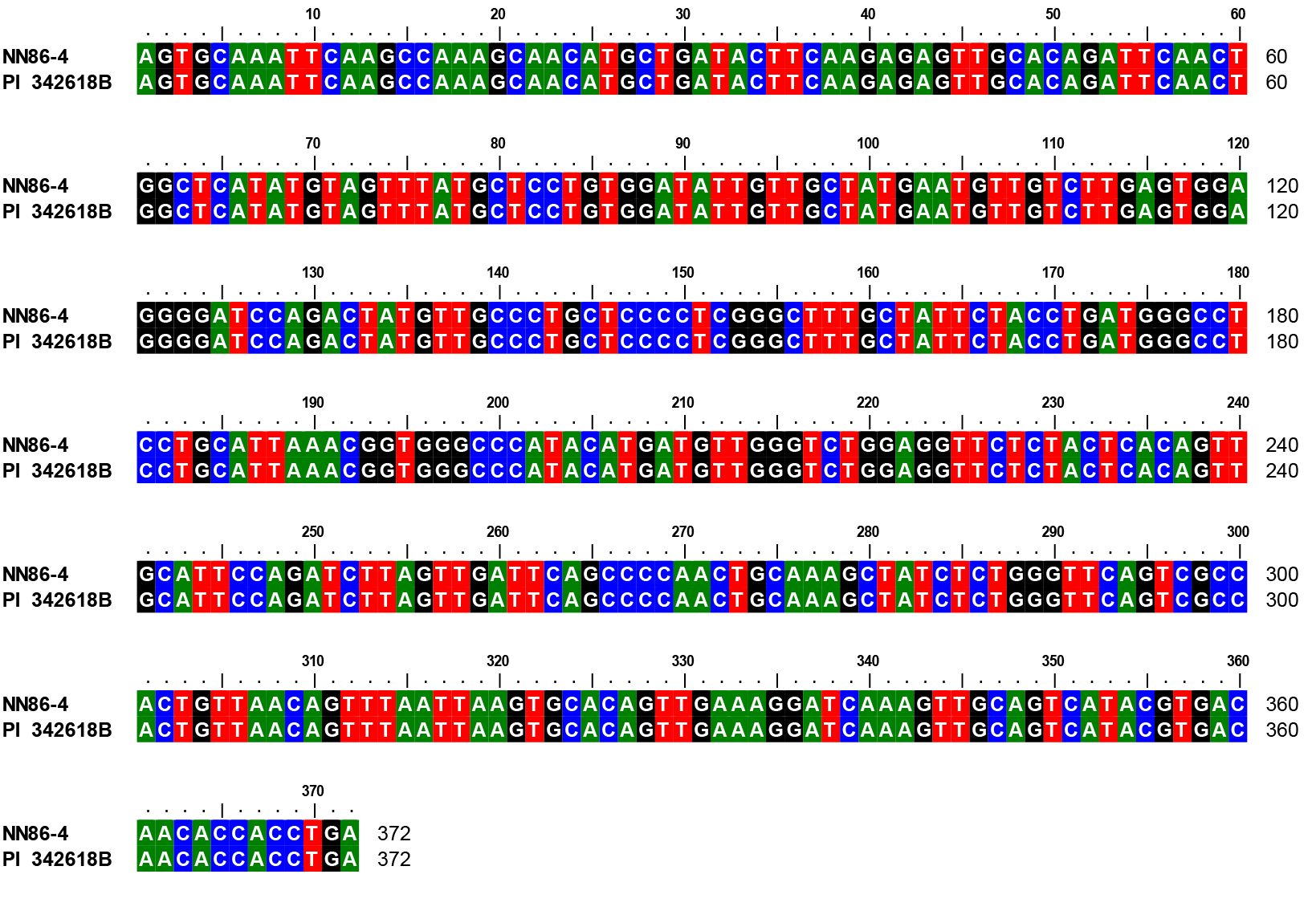

Supplement: Supplementary Figure 3 — DNA sequence alignment of the last exon of Glyma.01g240100 (Pd1) in two parents. [file Image_3.TIF]

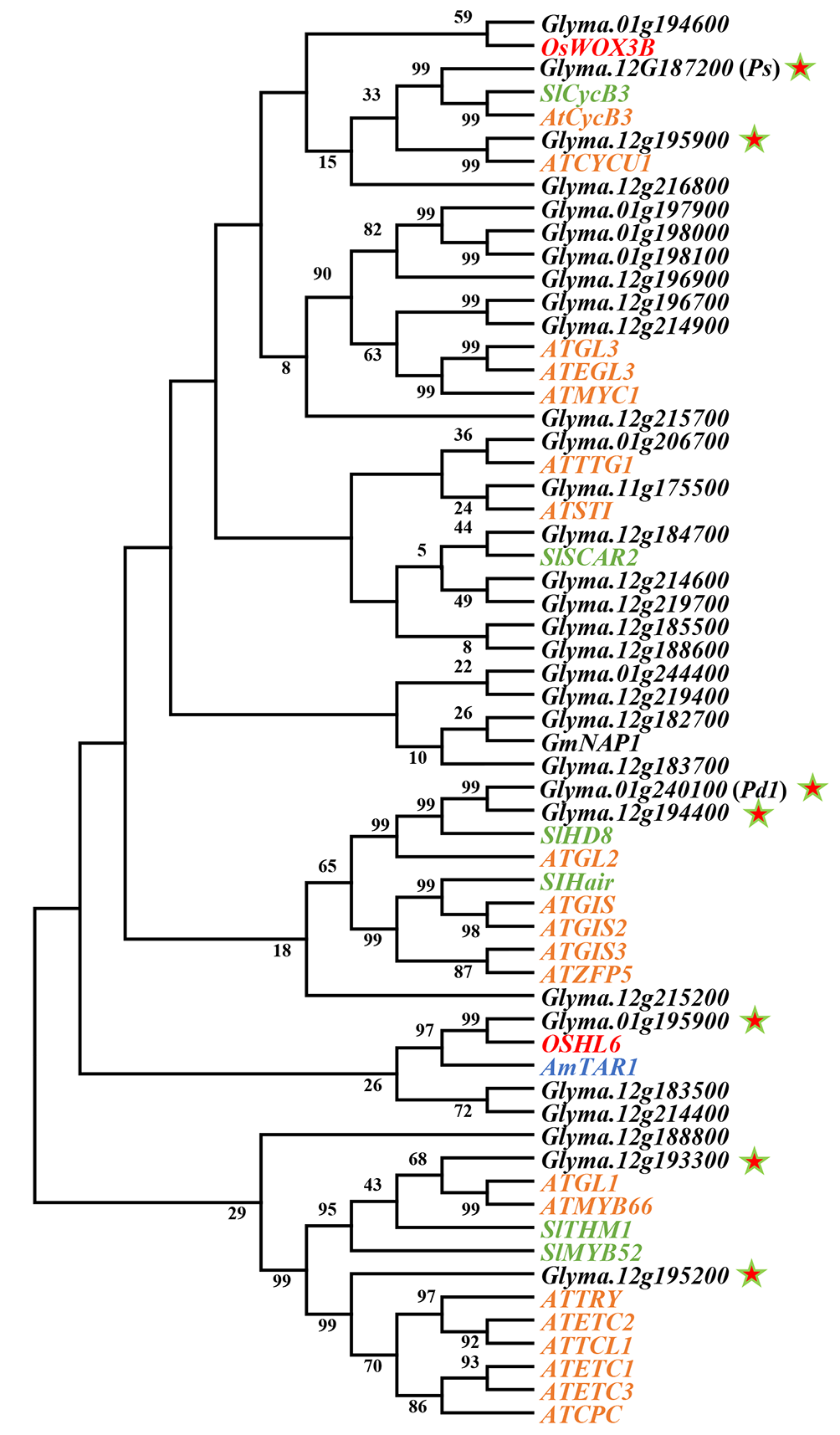

Supplement: Supplementary Figure 4 — Phylogenetic relationship between predicted candidate genes and their homologs based on literature. AT, Arabidopsis thaliana (thale cress); Glyma, Glycine max (Linn.) Merr. (soybean); Os, Oryza sativa (rice); Sl, Solanum lycopersicum (tomato); Am, Antirrhinum majus L. (Snapdragon). Gene names from different species are shown by different colors. Gene names with stars behind them indicate genes that can cluster with homologous genes. [file Image_4.TIF]
